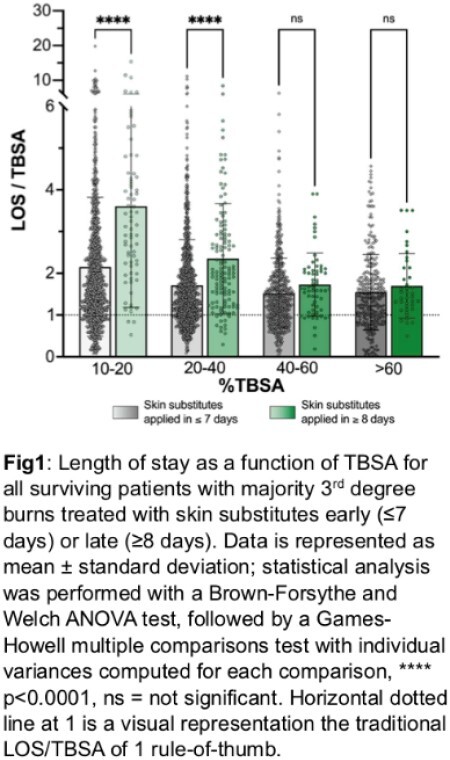# 87 Impact of Skin Substitute Use for Burns: An Analysis of the ABA National Burn Repository

**DOI:** 10.1093/jbcr/irae036.086

**Published:** 2024-04-17

**Authors:** Roselle Crombie, Claire Witherel

**Affiliations:** Connecticut Burn Center/Yale New Haven Health, Westport, Connecticut; Drexel/Penn, Princeton, New Jersey; Connecticut Burn Center/Yale New Haven Health, Westport, Connecticut; Drexel/Penn, Princeton, New Jersey

## Abstract

**Introduction:**

Skin substitutes provide unique utility for the management of thermal injuries over the last 20 years and have evolved into over 75 available technologies today. They are used for patients where insufficient autograft was lacking. Despite demonstrated long term safety and efficacy, use of skin substitutes remains controversial with respect to health economic metrics, including length of stay (LOS), complications, timing of application, number and types of procedures, resource use, and depth of burn. Clinical studies investigate skin substitute use in smaller burns (~20% TBSA) or in some anatomical regions (face, neck), but few address impact within a large national registry inclusive of all burn sizes and locations. The goals of this study were to investigate skin substitutes use for burns over the last 13 years and to elucidate where skin substitutes have the most utility in the burn care continuum.

**Methods:**

NBR data from 2008-2021 were analyzed (n = 388,775 patients). Surviving patients treated with a dermal regenerative graft during their care were identified via ICD-9 and ICD-10 codes (n=41,785 patients) and to show all surviving patients only and eliminating patients with data missing (n=32,428). Metrics including patient demographics (age, sex, race, comorbidities, burn degree, burn location/area) and outcome measurements (length of stay (LOS), total body surface area (TBSA) (2nd, 3rd, and combined), complications, resources, days in the ICU, number of procedures, number of excisional and non-excisional debridements). Additional analyses included determining the percentage of 2nd and 3rd degree burns (normalizing against total) and normalizing patients’ LOS per TBSA and investigating differences between skin substitute patients where their treatment was applied early (less than or equal to 7 days) or late (greater than or equal to 8 days).

**Results:**

Patients with the majority of their burn being 3rd degree and their skin substitutes applied within the first 7 days of admission, particularly for 10-20% TBSA and 20-40% TBSA were associated with a significantly lower LOS/TBSA compared to the same patients who received their skin substitute 8 days or later. Skin substitute use was associated with LOS/TBSA over 1.5 for all size burns when applied early and over 1.7 when applied late (Fig1).

**Conclusions:**

This study illustrates the first analysis of the ABA NBR investigating skin substitute use in burns, including high TBSA burns. Previous studies have limitations and overestimation of the traditional rule-of-thumb of LOS/TBSA near 1, including confounding variables such as age, complications, inhalation injury, and depth of burn. This study also shows that the care algorithm should be considered in outcome measures; future analysis will investigate this patient cohort to further understand the clinical and health economic implications of skin substitute use for burns.

**Applicability of Research to Practice:**

Knowing when to apply a skin substitute